# Increased uptake of early initiation of antiretroviral therapy and baseline drug resistance testing in San Francisco between 2001 and 2015

**DOI:** 10.1371/journal.pone.0213167

**Published:** 2019-03-14

**Authors:** Hong-Ha M. Truong, Sharon Pipkin, Robert M. Grant, Teri Liegler, Kara J. O’Keefe, Susan Scheer

**Affiliations:** 1 Department of Medicine, University of California, San Francisco, United States of America; 2 Gladstone Institute of Virology and Immunology, San Francisco, United States of America; 3 Department of Public Health, San Francisco, United States of America; Brigham and Women's Hospital, UNITED STATES

## Abstract

**Background:**

Early initiation of antiretroviral therapy (eiART) can improve clinical outcomes for persons with HIV and reduce onward transmission risk. Baseline drug resistance testing (bDRT) can inform regimen selection upon subsequent treatment initiation. We examined the uptake of eiART and bDRT within 3 months and 30 days of HIV diagnosis.

**Methods:**

We analyzed a population-based sample from the San Francisco Department of Public Health HIV/AIDS Case Registry of newly-diagnosed HIV/non-AIDS individuals between 2001 and 2015 who received care at publicly-funded facilities (N = 3,124).

**Results:**

Uptake of eiART within 3 months of diagnosis increased significantly from 2001 to 2015 (p<0.001), peaking at 74% in 2015. bDRT uptake also increased significantly (p<0.001), peaking at 55% in 2012. eiART uptake was observed to be significantly associated with gender, age, race/ethnicity and transmission risk. There were no significant differences observed in demographic and risk characteristics of persons receiving bDRT in the more recent years. Of 990 persons diagnosed between 2010 and 2015, eiART uptake within 30 days of diagnosis increased from 13% to 38% (p<0.001); bDRT uptake increased from 35% to 39% but the change was not significant (p = 0.141).

**Conclusions:**

Observed increases in eiART and bDRT uptake from 2010 to 2015 may reflect the adoption of treatment as prevention and a local public health policy statement in 2010 recommending treatment initiation at time of diagnosis irrespective of CD4 count. Concerns about stigma may underlie disparities in eiART, however such concerns would not bear as directly on a provider-initiated laboratory test like bDRT.

## Introduction

HIV treatment guidelines in the US have evolved greatly over the past decade. Historically, antiretroviral therapy (ART) initiation was based on CD4 T cell counts. Prior to 2007, ART initiation was recommended when CD4+ lymphocyte count (CD4 count) dropped below 200 cells/mm^3^. Treatment guidelines revisions were revised to recommend ART initiation at CD4 <350 in 2007 and CD4 <500 in 2009.[1]

Informed by clinical trials demonstrating starting ART early improves clinical outcomes for persons with HIV and reduces onward transmission risk, national treatment guidelines have recommended early initiation of ART (eiART) irrespective of CD4 count since 2012.[1–3] Prior to the national guideline revisions, the San Francisco Department of Public Health issued a policy statement in 2010 recommending ART initiation at time of diagnosis.[4]

Additionally, HIV-1 drug resistance testing (DRT) has long been recommended for the clinical management of patients failing treatment. When a regimen change is indicated due to virologic failure, DRT can guide the selection of drugs effective against the HIV strains a patient carries. Improvements in short-term virologic response to treatment when DRT results are available to clinician have been documented.[5]

DRT during early stages of infection has additional benefits. Baseline DRT (bDRT) upon diagnosis and prior to ART initiation can detect transmitted drug resistance mutants present early in the course of infection that decrease over time but might still persist at undetectable levels in the absence of ART.[6,7] bDRT was proposed as “reasonable to consider” in 2003 and first officially recommended in 2007.[1]

The present analysis examined the uptake of eiART and bDRT within both 3 months and 30 days of HIV diagnosis. We also assessed demographic, risk and clinical characteristics of newly-diagnosed persons with eiART and bDRT uptake.

## Methods

We analyzed a population-based sample of San Francisco residents newly-diagnosed with HIV/non-AIDS from 2001 through 2015 who received care at publicly-funded facilities. HIV/non-AIDS was defined as CD4 count ≥200 cells/mm^3^ and the absence of an AIDS-indicator condition at time of diagnosis. Receipt of care was defined as having either a CD4 count or an HIV viral load laboratory result. Data were obtained from the San Francisco Department of Public Health HIV/AIDS Case Registry, and the AIDS Research Institute (ARI) University of California, San Francisco (UCSF) Laboratory of Clinical Virology (LCV) which performs drug resistance testing for all local publicly-funded clinics.

Persons not ART-naïve at time of diagnosis were excluded from all analyses. ART initiation date was determined based on the prescription date indicated in the medical records. DRT date was based on the specimen collection date. Two definitions of eiART were used: 1) initiating ART within 3 months of diagnosis; and 2) initiating ART within 30 days of diagnosis. Two definitions of bDRT were used: 1) DRT within 3 months of diagnosis and prior to ART initiation; and 2) DRT within 30 days of diagnosis and prior to ART initiation. The timeframe (3 months or 30 days) was specified in each analysis. Since recommendations for eiART upon diagnosis began in San Francisco in 2010 and nationally in 2012, we assessed eiART and bDRT within 30 days of diagnosis for persons diagnosed between 2010 and 2015.

Demographic and risk characteristics included gender, age, race/ethnicity and HIV transmission risk category. Clinical characteristics included HIV diagnosis date, first DRT date and ART initiation date. Cases were characterized by diagnosis year and stratified by treatment guideline eras. Diagnosis years were grouped into eras based on treatment guidelines or when a significant local policy on ART was implemented. Era 1 spanned from 2001 through 2003, Era 2 from 2004 through 2006, Era 3 from 2007 through 2009, Era 4 from 2010 through 2012, and Era 5 from 2013 through 2015.

eiART and bDRT temporal trends and associations with demographic, risk and clinical characteristics were assessed by Chi-Square and Fisher’s Exact tests, and multivariable logistic regression. The study received approval from the UCSF Institutional Review Board. A waiver of informed consent was granted with the stipulation that all identifiable patient data, e.g., clinical results, and demographic and risk characteristics, would be maintained securely behind the mandatory data firewall of the San Francisco Department of Public Health.

## Results

A total of 3,124 persons newly-diagnosed with HIV/non-AIDS in San Francisco between 2001 and 2015 received care at publicly-funded facilities. These included 723 persons diagnosed in Era 1, 753 persons in Era 2, 658 persons in Era 3, 565 persons in Era 4 and 425 persons in Era 5. Demographic and risk characteristics, stratified by eras, are shown in Table 1. Across all eras, the largest proportion of individuals were males, whites, and men who have sex with men (MSM). In the first three eras (2001–2009), the largest proportion of individuals was between the ages of 30 to 39 years at diagnosis compared to other age groups; however, this shifted to ages 20 to 29 years in Eras 4 and 5.

**Table 1:** Demographic and risk characteristics of newly-diagnosed HIV/non-AIDS cases receiving care at publicly-funded facilities, by treatment guideline eras, San Francisco, 2001–2015 (N = 3,124).

	Total Cases N = 3,124			2001–2003 Era 1 N = 723		2004–2006 Era 2 N = 753		2007–2009 Era 3 N = 658		2010–2012 Era 4 N = 565		2013–2015 Era 5 N = 425	
	n	%	p-value	n	%	n	%	n	%	n	%	n	%
**Gender**			0.18										
Male	2,650	84.8		599	82.9	657	87.3	546	83.0	485	85.8	363	85.4
Female	332	10.6		89	12.3	71	9.4	71	10.8	57	10.1	44	10.4
Trans Female	142	4.6		35	4.8	25	3.3	41	6.2	23	4.1	18	4.2
**Age (years)**			<0.001										
< 20	48	1.5		13	1.8	11	1.5	8	1.2	8	1.4	8	1.9
20–29	911	29.2		158	21.9	215	28.6	204	31	184	32.6	150	35.3
30–39	1120	35.9		333	46.1	271	36.0	223	33.9	164	29.0	129	30.3
40–49	741	23.7		160	22.1	183	24.3	165	25.1	148	26.2	85	20.0
≥ 50	304	9.7		59	8.2	73	9.7	58	8.8	61	10.8	53	12.5
**Race/Ethnicity**			0.001										
White	1,404	44.9		347	48.3	353	47.3	299	45.0	250	43.0	155	36.3
Hispanic/Latino	785	25.1		148	20.5	196	26.1	164	25.2	152	27.3	125	28.9
Black	558	17.9		157	21.3	118	15.5	110	17.0	90	16.3	83	19.4
Asian/Pacific Islander	224	7.2		41	5.7	46	6.1	52	7.9	44	7.8	41	9.7
Other/Unknown	153	4.9		30	4.1	40	5.3	33	5.0	29	5.1	21	4.9
**HIV Transmission Risk**			<0.001										
Men who have sex with men	2,544	81.4		568	78.6	636	84.5	535	81.3	452	80.0	353	83.1
Persons who inject drugs	320	10.2		104	14.4	73	9.7	63	9.6	49	8.7	31	7.3
Heterosexual	182	5.8		33	4.6	30	4.0	47	7.1	44	7.8	28	6.6
Other/Unknown	78	2.5		18	2.5	14	1.9	13	2.0	20	3.5	13	3.1

**Fig 1:**
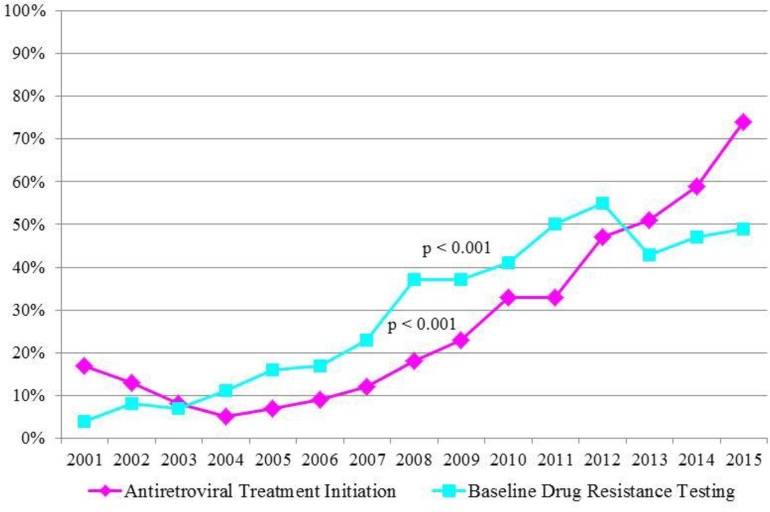
Antiretroviral therapy initiation and baseline drug resistance testing within 3 months of diagnosis, HIV/non-AIDS cases receiving care at publicly-funded facilities, San Francisco, 2001–2015 (N = 3,124).

Overall, there were 729 persons (23%) receiving eiART and 836 persons (27%) receiving bDRT within 3 months of diagnosis. The proportion of individuals by year is presented in Fig 1. Uptake of eiART and bDRT increased significantly from 2001 to 2015 (p<0.001); the proportion was 17% in 2001, decreased over the next 3 years to a nadir of 5% in 2004, then increased steadily to a peak of 74% in 2015. The proportion of individuals receiving bDRT also increased significantly from 2001 to 2015 (p<0.001); the low was 4% in 2001, gradually increased until a peak of 55% in 2012, and declined over the next 3 years to 49% in 2015.

**Fig 2:**
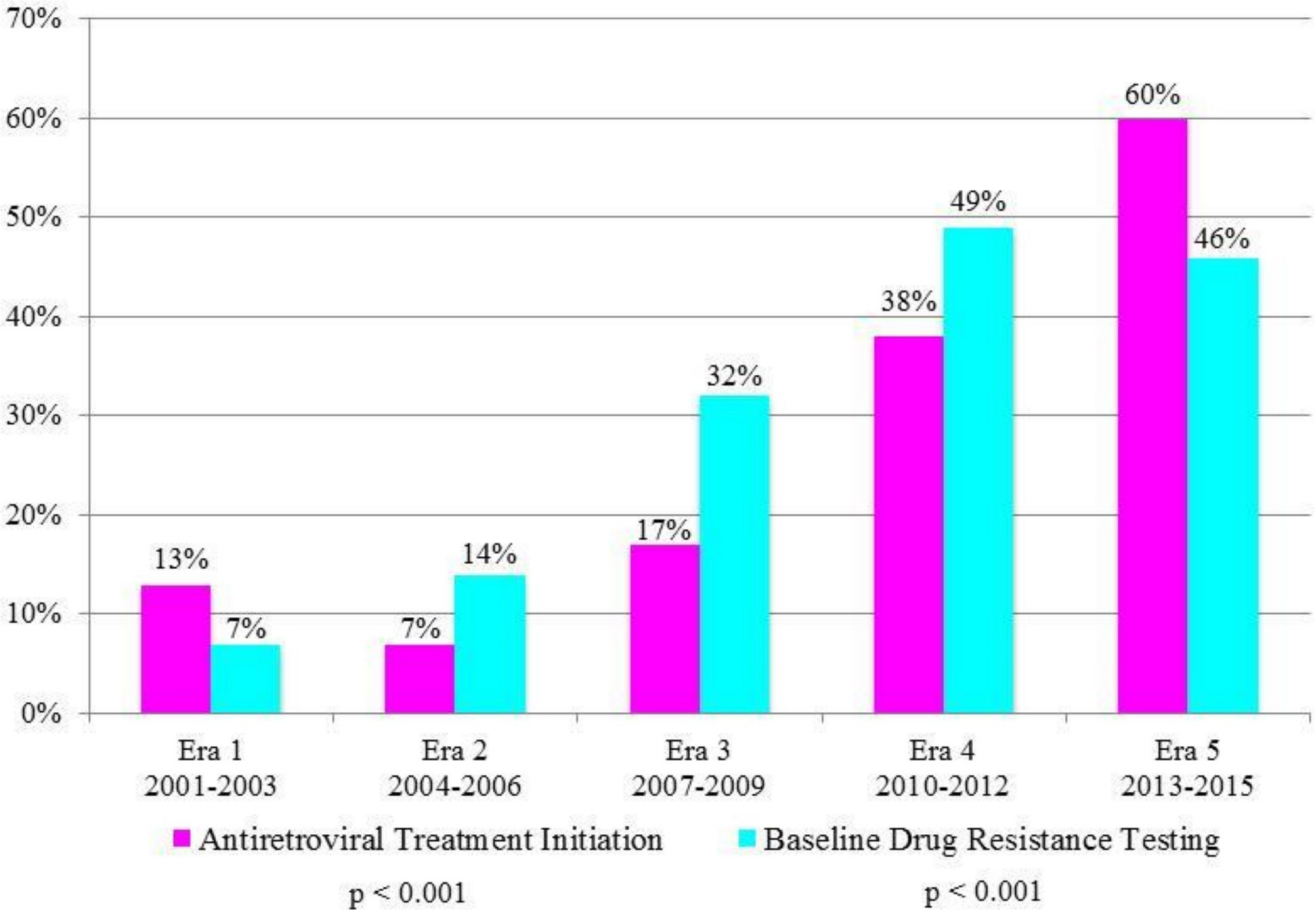
Antiretroviral therapy initiation and baseline drug resistance testing within 3 months of diagnosis, HIV/non-AIDS cases receiving care at publicly-funded facilities, by treatment guideline eras, San Francisco, 2001–2015 (N = 3,124).

The proportion of cases with eiART and bDRT within 3 months of HIV diagnosis stratified by eras is shown in Fig 2. The proportion of cases with eiART increased overall across eras (p<0.001), ranging from 13% (n = 91) in 2001–2003 to 60% (n = 257) in 2012–2015. The proportion of cases with bDRT also increased overall across eras (p<0.001), rising from a low of 7% in 2001–2003, to a high of 49% in 2010–2012, then decreasing slightly to 46% in Era 2013–2015. Across eras, among the 831 persons who received bDRT within 3 months of diagnosis, 391 individuals (47%) also received eiART within this timeframe. The proportion increased significantly (p<0.001), ranging from 27% in 2001–2003, decreasing to 18% in 2003–2006, rebounding to 29% in Era 2007–2009, rising to 54% in 2010–2012 and plateauing at 76% in 2013–2015.

Demographic and risk characteristics of persons receiving eiART and bDRT within 3 months of diagnosis, stratified by eras, are presented in Table 2. Males were more likely to receive eiART than females and trans females in 2004–2015. Persons <20 and 30–39 years old were more likely to receive eiART than those ages 20–29, 40–49 and ≥ 50 years in 2013–2015. Hispanics/Latinos and Asians/Pacific Islanders were more likely to receive eiART than whites and blacks in 2001–2003. MSM were more likely to receive eiART than persons who inject drugs (PWID), heterosexuals and persons with other/unknown risks in 2007–2009 and 2013–2015. In 2001–2003, males compared to females and trans females, and MSM compared to PWID, heterosexuals and persons with other/unknown risks, were more likely to receive bDRT. Blacks were less likely to receive bDRT than whites, Hispanics/Latinos and Asians/Pacific Islanders in 2001–2003. No significant differences in demographic and risk characteristics for bDRT in 2004–2015.

**Table 2:** Bivariate analysis of demographic and risk characteristics associated with persons newly-diagnosed with HIV/non-AIDS receiving care at publicly-funded facilities with early initiation of antiretroviral therapy (eiART) and baseline drug resistance testing (bDRT) within 3 months of diagnosis, by treatment guideline eras, San Francisco, 2001–2015 (N = 3,124).

Total Cases N = 3,124	2001–2003 Era 1 n = 723		2004–2006 Era 2 n = 753		2007–2009 Era 3 n = 658		2010–2012 Era 4 n = 565		2013–2015 Era 5 n = 425	
	%	p-value	%	p-value	%	p-value	%	p-value	%	p-value
**Early Initiation of Antiretroviral Therapy**										
**Gender**		0.313		0.021		0.042		0.005		0.035
Male	12.0		6.1		18.7		40.4		62.5	
Female/Trans Female	15.3		12.5		10.7		23.8		48.4	
**Age (years)**		0.343		0.806		0.307		0.370		0.009
< 20	15.4		9.1		12.5		12.5		87.5	
20–29	10.8		7.4		13.2		38.0		58.7	
30–39	14.1		6.6		18.4		41.5		70.5	
40–49	13.8		5.5		18.8		38.5		54.1	
≥ 50	5.1		9.6		24.1		31.2		47.2	
**Race/Ethnicity**		0.011		0.714		0.315		0.147		0.117
White	11.8		5.7		20.1		36.0		61.3	
Hispanic/Latino	18.2		7.7		13.4		41.5		67.2	
Black	7.6		9.3		15.5		31.1		50.6	
Asian/Pacific Islander	24.4		6.5		21.2		52.3		63.4	
Other/Unknown	6.7		7.5		12.1		37.9		47.6	
**HIV Transmission Risk**		0.218		0.120		0.028		0.083		0.046
Men who have sex with men	9.7		6.3		18.9		39.8		62.6	
Persons who inject drugs/Heterosexual/Other/Unknown	13.4		10.3		10.6		31.0		50.0	
**Baseline Drug Resistance Testing**										
**Gender**		0.043		0.927		0.942		0.656		0.760
Male	7.7		14.2		32.1		48.9		46.6	
Female	2.3		15.5		31.0		49.1		43.2	
Trans Female	0.0		16.0		34.2		39.1		38.9	
**Age (years)**		0.376		0.604		0.679		0.178		0.320
< 20	7.7		9.1		37.5		75.0		75.0	
20–29	7.0		13.5		34.8		50.0		48.0	
30–39	8.1		17.0		30.9		48.9		47.3	
40–49	5.0		12.0		28.5		41.9		40.0	
≥ 50	1.7		13.7		36.2		55.7		41.5	
**Race/Ethnicity**										
White	8.7	0.004	13.3	0.394	30.1	0.536	44.0	0.062	46.5	0.964
Hispanic/Latino	8.8		18.4		32.9		58.6		43.2	
Black	1.0		11.0		38.2		44.4		48.0	
Asian/Pacific Islander	9.8		15.2		26.9		50.0		46.3	
Other/Unknown	0.0		12.5		33.3		44.8		47.6	
**HIV Transmission Risk**		0.025		0.706		0.699		0.388		0.653
Men who have sex with men	8.1		14.3		32.0		48.9		46.5	
Persons who inject drugs	1.9		13.7		30.2		49.0		41.9	
Heterosexual	0.0		20.0		38.3		52.3		50.0	
Other/Unknown	0.0		7.4		23.1		30.0		30.8	

Of the 990 persons diagnosed between 2010 and 2015, 237 persons (24%) received eiART and 361 persons (36%) received bDRT within 30 days of diagnosis. The proportion of individuals receiving eiART increased significantly from 13% in 2010–2012 to 38% in 2013–2015 (p<0.001). The proportion of individuals receiving bDRT rose from 35% in 2010–2012 to 39% in 2013–2015 but the increase was not significant (p = 0.141). Demographic and risk characteristics of persons receiving bDRT are detailed in Table 3. Persons receiving eiART within 30 days of diagnosis were more likely to receive bDRT within 30 days of diagnosis than persons that did not in 2010–2012 (aOR = 2.3, p = 0.001) and 2013–2015 (aOR = 2.8, p<0.001). There were no significant differences observed in demographic and risk characteristics. Among the 361 persons receiving bDRT, 132 individuals (37%) also received eiART within this timeframe. The proportion increased steadily from 18% in 2010 to 79% in 2015 (p<0.001).

**Table 3:** Multivariable analyses of demographic and risk characteristics associated with baseline drug resistance testing within 30 days of diagnosis, newly-diagnosed HIV/non-AIDS cases receiving care at publicly-funded facilities, San Francisco, 2010–2015 (N = 990).

	2010–2012 Era 4 (n = 565)		2013–2015 Era 5 (n = 425)	
	aOR (95% CI)	p-value	aOR (95% CI)	p-value
**Gender**				
Male	Ref		Ref	
Female/Trans Female	0.7 (0.4–1.3)	0.26	0.6 (0.3–1.4)	0.27
**Age (years)**				
< 20	4.0 (0.9–18.9)	0.08	3.9 (0.7–21.2)	0.12
20–29	Ref		Ref	
30–39	0.9 (0.6–1.5)	0.73	0.9 (0.5–1.4)	0.55
40–49	0.7 (0.4–1.2)	0.18	0.8 (0.5–1.4)	0.50
≥ 50	0.8 (0.4–1.5)	0.40	0.8 (0.4–1.6)	0.48
**Race/Ethnicity**				
White	Ref		Ref	
Hispanic/Latino	1.3 (0.8–2.0)	0.27	0.9 (0.5–1.5)	0.64
Black	1.0 (0.6–1.8)	0.97	1.1 (0.6–1.9)	0.82
Asian/Pacific Islander	0.8 (0.4–1.6)	0.48	1.1 (0.5–2.3)	0.79
Other/Unknown	1.4 (0.6–3.2)	0.41	1.3 (0.5–3.5)	0.56
**HIV Transmission Risk**				
Men who have sex with men (MSM)	Ref		Ref	
Non-MSM	1.2 (0.7–2.2)	0.46	0.9 (0.5–1.9)	0.85
**ART initiation w/in 30 days of diagnosis**				
Yes	2.3 (1.4–3.9)	0.001	2.8 (1.8–4.4)	<0.001
No	Ref		Ref	
Missing/None	0.5 (0.3–0.8)	0.002	0.8 (0.4–1.4)	0.41

## Discussion

The proportion of persons newly-diagnosed with HIV/non-AIDS who initiated ART within 3 months of diagnosis increased significantly from 2004 through 2015. The substantial increase in eiART uptake observed in the more recent years likely reflect adoption of revised local and national guidelines, as well as the strategy of treatment as prevention.[2,3,8,9] The decrease seen in 2002 through 2006 may stem from concerns during that time regarding toxicities associated with ART. Disparities observed from 2004 through 2015 were driven primarily by the lower uptake of eiART among women, blacks, persons who inject drugs, heterosexuals, and other non-MSM groups. These groups are affected by multiple layers of stigma related to HIV status, race, ethnicity, sexual practices, gender, and substance use. Such compounded stigma is a substantial barrier to health care engagement.[10]

ART has been shown to increase survival and decrease morbidity, and the 2010 San Francisco universal treatment policy was influenced by findings released the previous year demonstrating lower mortality risk among patients initiating ART at higher CD4 counts.[8,9] However, concerns about side effects, the unknown impact of long-term use, and the costs have been found to influence the ART initiation decision for some providers and their patients.[11]

bDRT within 3 months of diagnosis increased steadily from 2004 through 2012, with a slight decline in 2013 to 2015, always remaining less than 40%. bDRT was less common in the early part of the decade, likely because the national guidelines at the time did not include bDRT. The finding that fewer than half of cases had bDRT was unexpected, especially in the more recent years when baseline testing recommendations had been placed for several years and testing had become available to San Francisco residents regardless of insurance coverage. Potential barriers to resistance testing include lack of familiarity with how resistance testing is ordered, especially in testing centers where HIV is diagnosed but treatment is not routinely provided. Financial barriers to resistance testing would still occur if the person was not a resident of San Francisco or if they migrated out of San Francisco before resistance testing was ordered. Ethnic disparities in treatment access and resistance testing were present in 2001–2003 and lessened in the following eras as treatment and resistance testing scaled up. While eiART involves multiple opportunities for stigma to create disparities, bDRT is a laboratory test that poses little additional risk of stigma or unintended disclosure.

The proportion of individuals receiving both bDRT and eiART within 3 months of diagnosis was highest in 2013–2015, which likely reflects the combined impact of revisions to the treatment and drug resistance testing guidelines. Both eiART and bDRT within 30 days of diagnosis increased between 2010 and 2015. The increase in bDRT was driven primarily by the corresponding increase in eiART. Local and national guideline revisions in recent years recommending treatment initiation upon diagnosis likely propelled the observed increases.

Even for cases receiving bDRT who did not initiate treatment early, baseline genotyping can inform regimen selection upon subsequent ART initiation because the abundance of resistant viruses may decline over time in the absence of selective drug pressure but may be still present in viral archives. Upon ART initiation, even low levels of resistant viruses may increase risk of treatment failure.[12–13] bDRT enables clinical documentation of resistant viruses before they revert and are overgrown by wild-type viruses. In addition, previous studies suggest bDRT may be cost-effective by optimizing regimens and thereby increasing rates of durable suppression, particularly in settings which have increasing prevalence of transmitted drug resistance, such as San Francisco.[14–16]

Associations observed among the study sample may differ from individuals receiving care at private facilities. Nonetheless, since publicly-funded facilities provide care for three-quarters of newly-diagnosed cases annually, these results are representative of a majority of the recently-diagnosed HIV population in San Francisco.[17] Data for cases with bDRT conducted at other laboratories would not be reflected in this analysis. However, since the ARI UCSF LCV conducts most of the DRT for publicly-funded facilities in San Francisco, data for the vast majority of this study population were available.

Both eiART and bDRT were adopted by local clinical practices in San Francisco as early as 2004, before the U.S. treatment guidelines included these recommendations on a national basis. Further analyses are needed to evaluate the benefits of the combined practice of eiART and bDRT in ultimately improving clinical outcomes.
